# Diphtheritic Paralysis in Children

**Published:** 1895-01-05

**Authors:** G. A. Sutherland

**Affiliations:** Physician to the North London Consumption Hospital, Registrar to the Paddington Green Children's Hospital


### MEDICAL PROGRESS AND HOSPITAL CLINICS.

[*The Editor will be glad to receive offers of co-operation and contributions from members of the profession. All letters should be addressed to* THE EDITOR, THE LODGE, PORCHESTER SQUARE, LONDON, W.]

### DIPHTHERITIC PARALYSIS IN CHILDREN.

By G. A. SUTHERLAND, M.D., M.R.C.P., Physician to the North London Consumption Hospital, Registrar to the Paddington Green Children's Hospital.

Diphtheria is associated with a form of paralysis which commences usually from two to three weeks after the subsidence of all acute symptoms, and the cessation of membranous formation. The gravity of this paralysis cannot be exaggerated, because of the danger of cardiac or respiratory failure which is always present. Hilton Fagge records a case of paraplegia which was admitted to hospital under his care, and the patient—a girl who had been running about the wards—died suddenly within a few days of admission. It was then discovered that she had recently had diphtheria, and it was recognised when too late what the nature of the paraplegia had been. A patient of my own, who was convalescent from diphtheria but had paralysis of the soft palate, was taken out of bed to be weighed. Within a few hours symptoms of cardiac failure appeared, the pulse rate increased rapidly to 200 beats per minute, and the boy died the same night. Another patient was sent into hospital with well-marked symptoms of diphtheritic paralysis, including cardiac irregularity and weakness. He died suddenly within two hours of admission from cardiac syncope. I mention these cases because by some the affection is regarded as a slight one, to be treated by change of air and plenty of exercise; but experiences like the above indicate very clearly that special precautions must always be taken. In many cases the diagnosis is rendered more difficult by the fact that there is no history of previous diphtheria, or even sore throat. Hence it has been argued that the series of clinical symptoms embraced under the term diphtheritic paralysis may be developed quite apart from diphtheria, as the result of other forms of sore throat or other acute diseases. This question has not been definitely settled, but the amount of evidence in favour of the latter theory is extremely small as compared with that supporting the view that all such cases are diphtheritic in origin. Many slight cases of diphtheria are followed by severe and extensive paralysis, probably owing to the fact that treatment of the primary disease has not been thorough or prolonged.

**Signs and Symptoms.**—The onset of paralytic symptoms is usually gradual, and advice is seldom sought until they have been in existence for two or three weeks, so that, generally speaking, from four to five weeks have elapsed since the primary attack. The initial change, in most cases, is an alteration in the voice, which becomes nasal in character, owing to paresis of the soft palate and consequent incomplete closure of the posterior nares in phonation. During the act of swallowing there may be regurgitation through the nose, at first only of liquids, but later of solids also. On inspection the soft palate may in the the early stages be seen to move freely during respiration, but later the paresis is changed into paralysis, and the uvula hangs motionless. In some cases there is loss of sensation in the palate and throat. The muscles of the throat and epiglottis may also be affected, and there develops an increasing difficulty in swallowing, with choking from the entrance of food into the larynx. The larynx itself may be involved, as manifested by a difficulty in phonation, the words being, as it were, jerked out with an effort, and by a peculiar tonelessness of the voice, and by a noiseless and ineffectual cough.

The above conditions in varying degrees are common to almost all cases of diphtheria. The parts affected correspond with the situations of the membranous deposit in the acute attack, and the symptoms in many cases are limited to these regions. In others the paralysis spreads to parts which have been entirely unaffected in the course of the primary disease. It is impossible to lay down the exact order in which further developments will appear, but special note is to be made of the state of the eyes, the lower extremities, and the back. Loss of accommodation is frequently present, so that the patient is unable to read, and suffers from diplopia. The pupillary reaction to light is present, but there is no contraction on accommodation. When at rest the pupils are rather larger than under normal conditions, and in some cases are considerably dilated. These changes are dependent on paralysis of the third nerve, in its branches to the ciliary and internal recti muscles. The external recti are not infrequently affected, first on one side and then on the other, with consequent internal strabismus. In the early stages the condition is merely one of paresis, and is not constantly present, so that even with a distinct history of squinting it may be difficult to detect any change in the eyes. As the disease progresses the paralysis becomes more marked and more constant. Weakness of the lower limbs may be the most striking symptom in diphtheritic paralysis, and loss of walking power follows, so that the patient reels from side to side as if tipsy, the limbs being at the same time dragged along. The muscles become soft and flabby, and wasting is present. A similar condition may exist subsequently in the arms, with loss of muscular and co-ordinating power. The spinal muscles are occasionally affected. The head may fall forwards from paralysis of its muscular supports, or the patient may be unable to sit up from weakness of the lower spinal muscles.

A third series of symptoms, in which the vital centres or the nerves proceeding from them are involved, and of which the prognosis is very grave, must next be considered. These are apt to appear suddenly in the course of an ordinary attack of diphtheritic paralysis, and the gastric, the respiratory, and the cardiac centres would appear to be the parts specially involved, for which reason Dr. Leonard Guthrie describes them as "bulbar crises." Minor attacks may be present and pass off in a few hours, but as a rule there is a rapid increase in the symptoms, and in many cases a fatal termination ensues. It is of special importance, therefore, to note the earliest signs of an attack, so that suitable treatment may be adopted. Some muscular act on the part of the patient, such as walking, sitting up in bed, &c., is not infrequently the immediate precursor of one of these seizures. Sickness and vomiting coming on without any relation to meal-times, and accompanied by severe abdominal pain, being a danger signal which should not be overlooked. A certain amount of cardiac weakness is present in all patients who have passed through an attack of acute diphtheria. During the subsequent paralysis the heart may be still further weakened by the involvement of the cardiac branches of the vagus. Præcordial pain, rapidity, irregularity, and intermittency of the cardiac action, with weakness of the first sound, are the chief signs of this condition. The dangers are sudden death from syncope, and, in those cases in which the pulse rate has run up to 150 or 200 beats per minute, a more gradual termination from cardiac exhaustion. In other cases an abnormal slowness of the heart has been present, and this condition is also one of danger. The respiratory muscles are usually involved in the paralysis when the heart is markedly affected. In the early stages there is no sign of dyspnœa while the patient is at rest, but at intervals a long drawn or sighing inspiration is present. On examination one can usually detect impaired action of the diaphragm, and possibly also of the intercostal muscles. As the paralysis increases dyspnœa becomes more marked, the respiratory muscles act more and more feebly, pulmonary œdema supervenes, and the patient dies asphyxiated.

The sensory changes in diphtheritic paralysis are by no means so frequent or so marked as the motor. Numbness, tingling, and aching may be present in the limbs. The amount of wasting of the muscles varies with the degree and duration of the paralysis. In some cases it is extreme, and the patient is unable to perform even the most simple voluntary act. Such a condition, however, is very rare except in neglected cases.

**Diagnosis.**—In those cases in which the patient has been medically attended during and after an attack of diphtheria, there is no difficulty in recognising the symptoms of paralysis and referring them to their real cause. But in many cases the acute disease has been quite overlooked, and the first signs of illness noted have been an alteration in the speech, or a tendency to walk unsteadily, or it may be merely an impaired state of health. Attention must then be directed to a history of recent illness and to the signs of disease actually present. As regards recent illness, a history of sore throat, or difficulty in swallowing, or swelling in the neck, or nasal discharge, or epistaxis, or even malaise for a couple of days may prove valuable presumptive evidence of diphtheria. Enquiry must be made as to whether any other members of the family or residents in the same house had suffered from sore throat. It must also be remembered that an attack of diphtheria affecting other parts than the throat, for example the eye, or the vulva, may lead to all the paralytic spmptoms described above.

The most frequent evidences of paralysis in the early stages are a nasal tone of the voice, a difficulty in swallowing, and an unsteadiness in walking. In addition to these a considerable amount of languor is usually present, so that the patient is more inclined to seek quiet and rest than to run about. In the later stages of the affection this languor usually develops into complete apathy from general weakness. On examination of the throat, one can often detect irregularities in the tonsils, and traces of recent acute inflammation. A very important diagnostic sign, and one which should always be looked for in doubtful cases, is loss of the knee jerk. The patellar reflex is frequently lost during the attack of diphtheria, and it is almost invariably absent before any other nervous sequelæ have appeared. Further, the knee jerk is not usually restored until some months after the patient's health has been completely restored in every other respect.

